# Age-dependent accumulation of tau aggregation in *Caenorhabditis elegans*

**DOI:** 10.3389/fragi.2022.928574

**Published:** 2022-08-19

**Authors:** Wendy Aquino Nunez, Benjamin Combs, T. Chris Gamblin, Brian D. Ackley

**Affiliations:** ^1^ Laboratory Department of Molecular Biosciences, The University of Kansas, Lawrence, KS, United States; ^2^ Department of Translational Neuroscience, Michigan State University, Grand Rapids, MI, United States; ^3^ Department of Neuroscience, Developmental and Regenerative Biology, University of Texas San Antonio, San Antonio, TX, United States

**Keywords:** *C. elegans*, tau, tauopathy, microtubule associated proteins, neuronal aging

## Abstract

Aging is the primary risk factor for Alzheimer’s disease (AD) and related disorders (ADRDs). Tau aggregation is a hallmark of AD and other tauopathies. Even in normal aging, tau aggregation is found in brains, but in disease states, significantly more aggregated tau is present in brain regions demonstrating synaptic degeneration and neuronal loss. It is unclear how tau aggregation and aging interact to give rise to the phenotypes observed in disease states. Most AD/ADRD animal models have focused on late stages, after significant tau aggregation has occurred. There are fewer where we can observe the early aggregation events and progression during aging. In an attempt to address this gap, we created *C. elegans* models expressing a GFP-tagged version of the human tau protein. Here we examined how tau-gfp behaved during aging, comparing wild-type tau (hTau40), a disease-associated mutation (P301S), and an aggregation-prone variant (3PO). We measured age-dependent changes in GFP intensity and correlated those changes to normal aging in the nematode. We found differences in tau stability and accumulation depending on the tau variant expressed. hTau40GFP and P301SGFP were localized to axons and cell bodies, while 3POGFP was more concentrated within cell bodies. Expression of 3POGFP resulted in decreased lifespan and variations in locomotor rate, consistent with a pathological effect. Finally, we found that the human tau interacted genetically with the *C. elegans* ortholog of human tau, *ptl-1,* where the loss of *ptl-1* significantly accelerated the time to death in animals expressing 3PO.

## Introduction

Aging is the main risk factor for Alzheimer’s disease (AD) and other AD-related disorders (ADRD). AD is the most common form of senile dementia and affects over 6.5 million people (Rajan et al., 2021). The population of people 65 years and older is projected to double by 2050 (Nations, 2019). These statistics demonstrate the urgency to understand how aging increases the chances of developing neurodegenerative disorders (e.g., AD and ADRDs) and how the presence of aggregated proteins accelerates aging and increases the mortality rate.

Normal aging is characterized by a decline in protein homeostasis (Taylor and Dillin, 2011; López-Otín et al., 2013). The decline in cellular processes that maintain proteostasis can lead to protein aggregation (Ben-Zvi et al., 2009; Huang et al., 2019). Post-mortem brains of individuals diagnosed with AD and ADRDs have a high concentration of intracellular neurofibrillary tangles (NFTs) composed of the protein tau. Most people will spontaneously develop oligomeric complexes distinct from aggregated tau in addition to NFTs as they age (Huang et al., 2019), and at least one study has shown that NFTs correlate with normal, age-related cognitive decline (Guillozet et al., 2003). However, individuals with AD or ADRDs will develop a higher concentration of NFTs, especially in regions of the brain where synaptic loss and neuronal death can be observed (Chiu et al., 2017; Ziontz et al., 2019; Chen et al., 2021). Therefore, questions remain about the differences in tau aggregation during normal aging versus disease, and how age-related declines might be accelerated in the diseased state.

There are several animal models for AD and other ADRDs, with many that focus primarily on the aggregation of amyloid-ß (Edelstein-keshet and Spiros, 2002; Proctor and Gray, 2010; Puri and Li, 2010). Recent research, however, has highlighted the importance and complexity of the protein tau in these disorders. Age-related changes in tau have been shown to impair bioenergetics (reviewed in (Grimm, 2021)), autophagy (Chatterjee et al., 2021), calcium consumption (Datta et al., 2021), and other cellular events. In addition, structural studies have shown that tau adopts different conformations in disease that might have different disease causation capabilities (Oakley et al., 2020; Scheres et al., 2020; Shi et al., 2021). There is still a need for new *in vivo* models capable of visualizing tau protein dynamics, to measure the effect of aging, genetics, cell types or other environmental variables on aggregation.

Here we describe a model to visualize tau during aging in a *C. elegans* model. The nematode *C. elegans* has been used as a model organism to identify mechanisms that regulate lifespan (Kenyon, 2010; López-Otín et al., 2013). Several studies have highlighted the relationship between proteome remodeling, protein aggregation, and normal aging in the nematode (David et al., 2010; Ciryam et al., 2013; Walther et al., 2015; Huang et al., 2019). One recent study showed long-lived nematode mutants had a delayed onset of declining health, motivating a better understanding of how the aging process induces age-associated declines (Statzer et al., 2022). Animal models of tau aggregation have consistently reported a decrease in lifespan attributable to the overexpression of human tau (Wittmann et al., 2001; Sealey et al., 2017; Higham et al., 2019; Macdonald et al., 2019). Similarly, nematodes expressing human tau also showed a reduced lifespan and verified the presence of aggregated tau (Kraemer et al., 2003; Pir et al., 2016). Here we found that expression of gfp-tagged human tau reduced nematode lifespan, with a greater reduction associated with an aggressively aggregating version of tau (3PO). This decrease in lifespan was enhanced by a mutation that deleted the gene, encoding the endogenous microtubule associated protein, PTL-1, suggesting it was protective in this context.

## Materials and methods

### Strains and genetics

N2 (var. Bristol) was used as the wild-type reference strain in all experiment. Strains were maintained at 18–22°C, using standard maintenance techniques as described (Brenner, 1974). Strains used in this report include: RB809 [*ptl-1(ok621)*], NW1229 *evIs111* [P*rgef-1*:GFP] (Altun-Gultekin et al., 2001), EVL1654 *lhIs94* [P*rgef-1*:hTau40GFP], EVL1644 *lhIs92* [*Prgef-1*:hTau40-P301SGFP], and EVL1372 *lhIs84* [P*rgef-1*:3POTauGFP]. To create tau transgenes DNA was injected at the following concentrations: *lhEx274* (P*rgef-1::*hTau40GFP 5 ng/μl; *Pstr-*1:GFP 5 ng/μl); *lhEx646* (P*rgef-1::*hTau40(P301S)GFP 5 ng/μl; P*myo-2*:RFP 5 ng/μl; *Pstr-*1:GFP 5 ng/μl); *lhEx327* (P*rgef-1::*3POTauGFP 5 ng/μl; P*str-1::gfp* 5 ng/μl). Transgenes were integrated using Trimethylpsoralen and UV as described (Najarro et al., 2012). Animals were synchronized by starvation hatching and then placed on *E. coli* (OP50) to initiate development.

### Whole-mount immunostaining

Anti-TAU staining was done using Bouin’s fixation as described (Nonet et al., 1997). The primary antibodies used in this study were: rabbit anti-TAU (1:1000, A00024-A Agilent), mouse anti-TAU (1:500, TNT1) (Kanaan et al., 2011; Combs et al., 2016). The secondary antibodies used were Alexa 488-labeled goat anti-rabbit and Cy3 goat anti-mouse at 1:500 dilution.

### Fluorescence microscopy and image analysis

Images were obtained using an Olympus FV1000 laser-scanning confocal microscope equipped with the Fluoview software. Z-stacked images were taken and exported to ImageJ (Schneider et al., 2012). Images were z-projected using max intensity, and ROI was drawn around the relevant areas of the tail (for GFP images) or the ventral nerve cord (for the immunostaining). The following measurement options were selected: area, mean, minimum, maximum, integrated pixel intensity, and raw integrated pixel intensity. A region of the image adjacent to the animal was used to assess the background, which was subtracted from the raw pixel density and then that value was divided by the area to arrive at a normalized value for the reported GFP fluorescence.

### Biochemical characterization

Nematodes were grown on standard NGM plates and maintained at 20°C. Gravid nematodes were harvested and washed off the plate using M9 buffer and collected in a 15 ml conical tube. Embryos were extracted using bleach solution and incubated for 5 min while rotating. Embryos were washed four times and resuspended in M9 buffer, where they were allowed to hatch overnight, in the absence of food. Starvation-arrested L1 worms were grown in liquid culture containing *E. coli* (HB101), cholesterol, and 1x HyClone Antibiotic-Antimycotic Solution (Thermo Scientific). Worms were maintained with constant shaking at 20°C and collected and washed in M9 buffer on days one and three of adulthood. A final wash with ice-cold RAB reassembly buffer supplemented with protease/phosphatase inhibitor cocktail (Thermo Scientific, PI78440) and 5 mM EDTA. 100mg of compact worms were then aliquoted into 1.5 ml tubes and stored at −80°C.

Sequential protein extraction was performed as in (Kraemer et al., 2003) with minor modifications. Briefly, tau fractions were obtained by resuspending 100 mg worm pellet in two times (wt/vol) high salt RAB reassembly buffer (G-Biosciences, PI53113) supplemented with a 1x final concentration protease/phosphatase inhibitor cocktail (Thermo Scientific, PI78440). The same volume (compact worm/beads) of ice-cold 0.5 mm glass Beads (BioSpec Products) was added to the tube. Worms were lysed using a BeadBug Microtube Homogenizer (Benchmark Scientific) at max speed for 5 s, four times with 20-s rest on ice in between each session. The solution was incubated on ice for 30 min then centrifuged at 4°C (20817xg) for 30 min. The supernatant was considered the soluble fraction. The remaining pellet was washed with ice-cold RAB buffer and extracted with RIPA buffer with EDTA (G-Biosciences, AAJ61529AK), by incubation on ice for 20 min and centrifugation at 4°C (20817xg) for 15 min. The resulting supernatant was considered the detergent-soluble fraction. The pellet was washed with ice-cold RIPA buffer and extracted with urea (30 mM Tris, 7M urea, 2M thiourea, 4% CHAPS (3-[(3-cholamidopropyl)dimethylammonio]-1-propanesulfonate), pH 8.5] (Pir et al., 2016), incubated for 15 min at room temperature and centrifuged at room temperature (RT) (20817xg) for 10 min. The resulting supernatant was considered the urea soluble fraction.

Protein fractions were resolved in a 10% polyacrylamide gel and transferred to a PVDF membrane (Immobilon, IPFL00005). The following antibodies were used: Monoclonal anti-tau Tau-5 (1:2000; Thermo Scientific), Tau-7 (1:2000; Millipore Sigma), Tau-12 (1:2000; Millipore Sigma), Anti-Alpha-Tubulin (1:10,000; Thermo Scientific), anti-mouse HRP-labeled secondary antibody (1:5000; Thermo Scientific). PVDF membranes were visualized using Licor Odyssey Fc Imager. Western blot images were transferred to PowerPoint for cropping, alignment and annotation; no post-acquisition adjustments were made to brightness or contrast.

### Survival analysis

50-late stage L4 were transferred to an OP50 seeded NGM plate and maintained at 20°C for the duration of the assay. Live animals were counted and moved to a fresh plate each day for the first 7 days of the assay and then every 2–3 days after the worms had stopped laying eggs. The number of dead worms was recorded. 2–5 independent experimental replicates were done per strain.

### Locomotion analysis

Nematode locomotion was analyzed using WormLab Tracking Software (MBF Bioscience). L4 nematodes were selected and grown on standard NGM plates spotted with *E. coli* (OP50). Animals were transferred daily onto new plates and maintained at 20°C. On the day of the assay, nematodes were transferred to a fresh NGM plate (without food) to eliminate the excess OP50 from the nematode’s cuticle. Individual worms were selected and placed in a new NGM plate (no food) and allowed to acclimate for 1 min before acquiring the video. A 1-min recording (11.1 frames per second) was captured using a CCD camera (Leica DFC 3000G) attached to a fluorescent stereo microscope (Leica M165 FC). Speed was measured on days 1, 3, 5, and 7 of adulthood.

### Statistics

Analyses were performed using GraphPad (9.0.0) or using R in RStudio (Team, 2019; Team, 2020). For GFP analysis an Interaction ANOVA model (day*tau variant) was calculated, and significantly different interactions were assessed using a Tukey’s HSD test, and a P-adjusted value of <0.01 was used to determine significance. A Log-rank (Mantel-Cox) analysis was used for the comparison of survival curves. A threshold for significance was established at *p* < 0.05 and is defined in the figure legend and noted with asterisks.

### Cloning and molecular biology

Tau variants were amplified by PCR from bacterial expression clones. The original hTau40 expression vector (pT7C) was described in (Carmel et al., 1996). P301S was generated from hTau40 in the pT7C vector using the QuikChange Site-Directed Mutagenesis Kit (Stratagene) following manufacturer’s protocols. 3PO tau was generated from hTau40 by making the following modifications: I278N + N279I + ΔK280 + V309Y + Y310V + ΔP312 + S341I + ΔE342, using the QuikChange Site-Directed Mutagenesis Kit (Stratagene), based on the previously described strategy for optimizing the spontaneous aggregation of tau (Iliev et al., 2006). PCR products were inserted into pCRII-TOPO (Invitrogen) using the manufacturer’s protocol. The entry clones were recombined into a P*rgef-1*:GW:*gfp::unc-54* 3′UTR destination vector (pCZ#599, a generous gift of the Jin lab) using LR recombinase (Invitrogen) using manufacturer’s protocol to generate pEVL415 (P*rgef-1*:*htau40::gfp::unc-54* 3′UTR), pEVL538 (P*rgef-1*:*3POtau::gfp::unc-54* 3′UTR), pEVL539 (P*rgef-1*:*htau40(P301S)::gfp::unc-54* 3′UTR).

### Protein alignments

The microtubule binding repeats from the 2N4R human MAPT protein (NP_001116538.2) were aligned to the microtubule binding repeats from PTL-1 (NP_001367354.1) using Clustal Omega (Sievers and Higgins, 2021). The sequences are annotated with known human variants with the potential pathogenicity as described by the ClinVar database (Landrum et al., 2020).

## Results

### Wild-type tau expression in neurons is well tolerated

Our understanding of tau biology has increased dramatically since it was discovered to be a part of the neurofibrillary tangles in AD (Grundke-Iqbal et al., 1986; Wood et al., 1986; Goedert et al., 1988; Wischik et al., 1988). We now know that mutations along the MAPT gene, which codes for tau protein, can cause various disorders called tauopathies (Rossi and Tagliavini, 2015). Genetic mutations, or external factors, can lead to heterogenous tau aggregates that give rise to different phenotypic spectrums (Oakley et al., 2020; Scheres et al., 2020; Shi et al., 2021). However, understanding the spectrum of initiating events that cause tau to transition from monomeric to oligomeric to larger aggregates, and the different mechanisms of how tau polymers cause disease has proven challenging (Orr et al., 2017).

**FIGURE 1:**
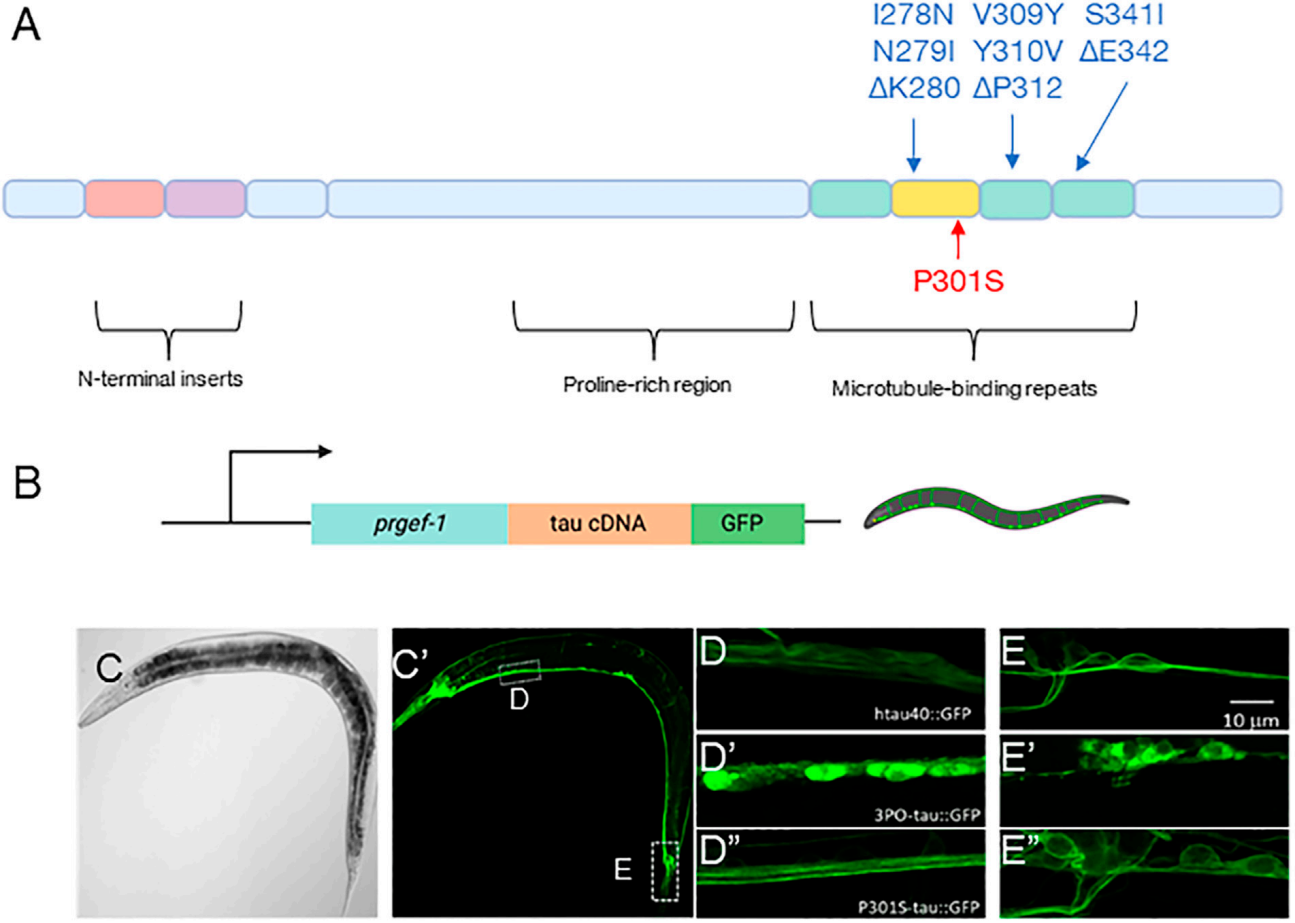
Pan-neuronal model of tau aggregation. **(A)** Schematic representation of the 2N4R tau isoform (hTau40) and the mutations used in this report. **(B)** A schematic representation of the DNA constructs injected (created with BioRender.com). **(C)** A transmitted light and **(C′)** fluorescent image of a day one adult animal expressing hTau40GFP. Magnified confocal images of the **(D)** ventral nerve cord and **(E)** tail regions of the animals in the wild-type hTau40, 3PO **(D’,E’)** and P301S **(D”, E”)** expressing animals. Note, the images are taken from regions indicated by the hashed boxes in **(C)**. **(D)** We found that in the ventral nerve hTau40 was evenly concentrated throughout axons, in contrast to **(D′)** 3POtau which was concentrated in the cell bodies. **(D”)** P301S was similar to the wild-type tau. **(E,E’,E”)** In the tail we observed similar results, with tau present through the cell bodies.

Animal models that mimic aspects of Alzheimer’s disease (AD) and other Alzheimer’ related disorders (ADRDs) are key to understanding the etiology of these diseases. Therefore, we set out to build an *in vivo* model to study tau using the nematode *C. elegans*. We created integrated transgenic animals expressing a GFP-tagged version of the longest human tau isoform (hTau40), pan-neuronally using the *rgef-1* promoter (Chen et al., 2011) (Figures 1A,B), because this promoter has been shown to have relatively stable expression throughout aging (Adamla and Ignatova, 2015). Additionally, we generated similar animals using tau variants, either P301S, which is a mutation associated with frontotemporal dementia (FTD), often used in research (Yoshiyama et al., 2007; Takeuchi et al., 2011), or 3PO, which is a lab construct that was optimized to spontaneously aggregate (Iliev et al., 2006) (Figure 1A). We found that, when DNA was injected at a relatively low concentration (5 ng/μl) expression of wild-type tau was well tolerated. That is, we did not observe any obvious defects in organismal morphology or impacts on survival during development, compared to animals expressing GFP alone under the same promoter as a negative control (*evIs111*) (data not shown). Thus, we used this concentration for all remaining experiments.

We observed tauGFP to be expressed throughout the entire nervous system of the nematode (Figure 1C’). In animals expressing wild-type tau, hTau40GFP appeared to be largely distributed through axons and neuronal cell bodies (Figures 1D,E). The P301S variant was like the wild-type protein (Figure 1D”, E”). In contrast, the 3PO variant was concentrated in cell bodies, with less material present in processes (Figure 1D’, E’). The appearance and localization of 3POtau is consistent with cell culture studies (Iliev et al., 2006). Based on these results, we concluded that we could observe tau localization inside *C. elegans* neurons.

### TauGFP distribution and appearance change in aging with aggregation

**FIGURE 2:**
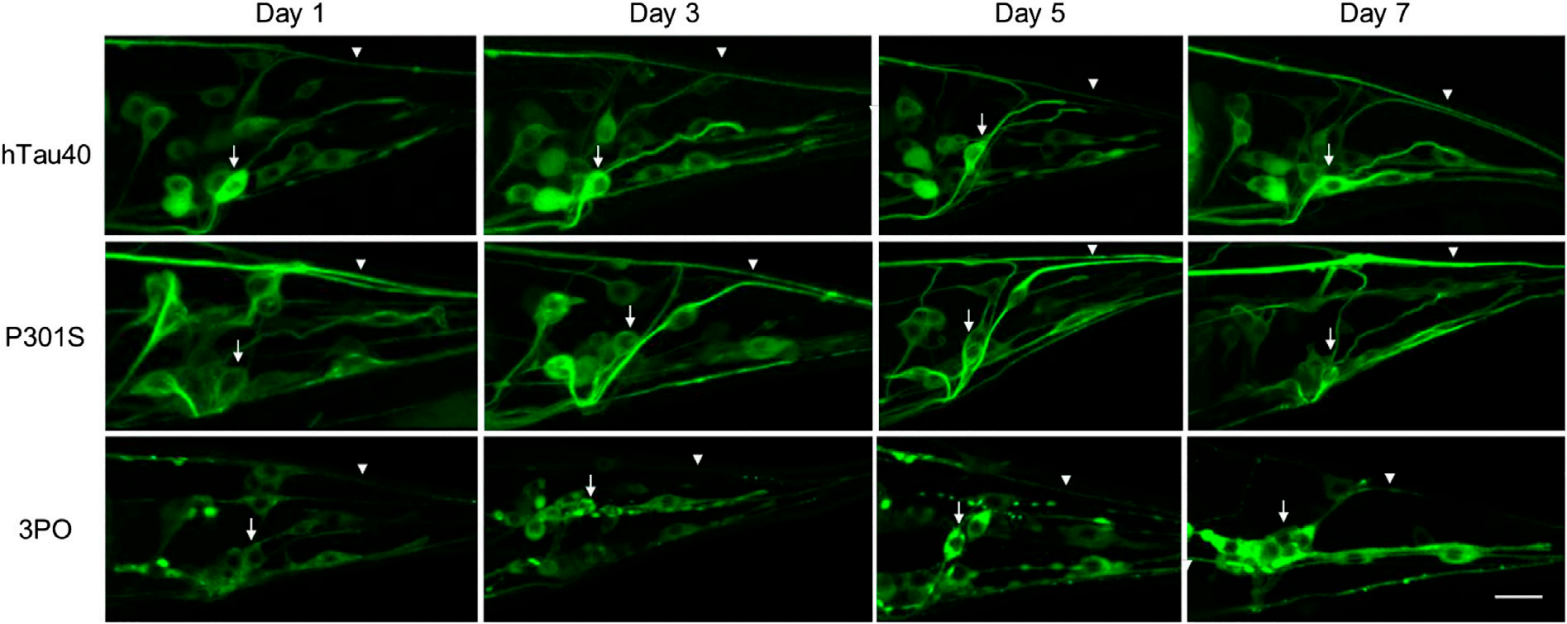
Age-dependent accumulation of tauGFP. We monitored TauGFP during aging in the animals. Across the first 7 days of adulthood, hTau40GFP was observed in cell bodies (arrows) and in axonal processes (arrowheads) consistently across aging. Compared to the wild-type we noticed that P301SGFP appears to be enriched in the axons (arrowheads), with a decrease in cell body fluorescence (arrows) during aging. 3POGFP was preferentially accumulated in cell bodies (arrows), with less fluorescence present in nerve processes (arrowheads). In addition, tau in the cell bodies in animals expressing 3POGFP appeared to be more clustered than those in animals expressing wild-type or P301S tau. Scale bar equals 10 μm.

**FIGURE 3:**
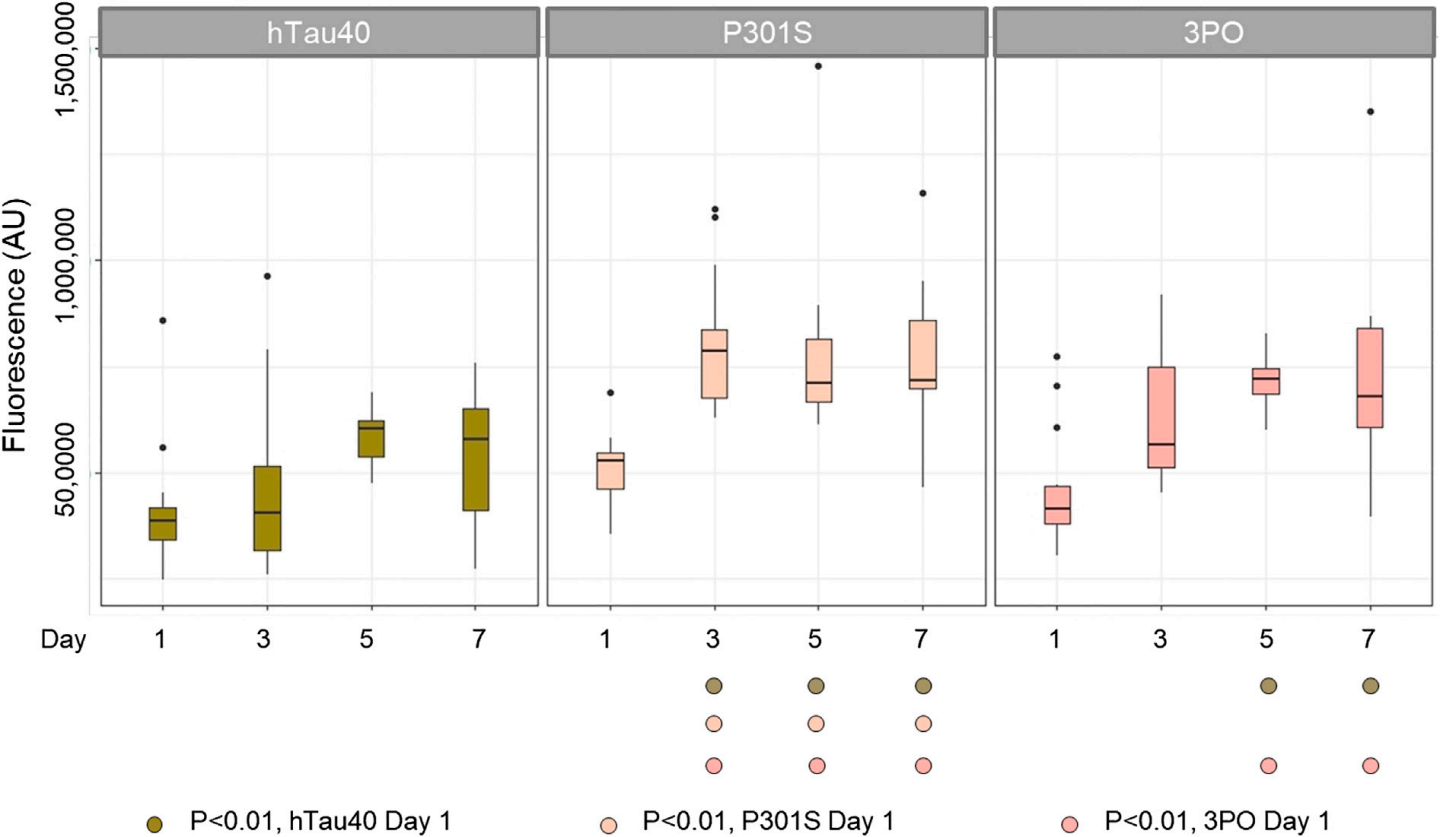
TauGFP is stable and accumulates in an age-dependent manner in neurons and axons. Day 1 nematodes strains have similar GFP intensity suggesting that the tau integrants have equivalent levels of expression. By day 3 (P301S) and day 5 (3PO) fluorescence was significantly increased, respectively, suggesting a significant buildup of tau while hTau40 remained statistically constant during the selected time points. ANOVA (Day*Variant) with Tukey post-hoc comparison. *N* = 12–15.

Aging is the main risk factor for AD and ADRDs, so we monitored changes in tauGFP during aging. We imaged neurons located in the tail of the animals on days one, three, five, and seven of adulthood (Figure 2). This region provided a consistent and well-defined location and avoided potential background effects of the intestinal autofluorescence, which naturally increases with age and stress. Quantifying fluorescence via image analysis, we found discrete age-dependent changes in tauGFP localization and quantity that was different, depending on the tau variant expressed (Figure 3). Qualitatively, the pattern of hTau40GFP was largely the same over the first week of adulthood exhibiting localization to cell bodies and axons alike. There were no significant differences in GFP intensity in hTau40-expressing animals as a function of age during the first week of adulthood (Figure 3).

Next, when we examined the changes in the disease-associated variant, P301SGFP, we observed a different pattern. While the distribution of GFP on day one appeared to be roughly equal between cell bodies and processes, as the animals aged, we noticed a qualitative enrichment in the processes and a relative depletion in cell bodies, a pattern not observed for hTau40GFP (Figure 2). When we quantified the total fluorescence, there was a significant increase in the fluorescence observed by day three (Figure 3). Thus, we conclude that the P301S variant preferentially localized to neuronal processes, compared to cell bodies. The increased intensity over time, suggests it may be more stable *in vivo* compared to hTau40 (Figure 3). For the 3POGFP animals we also observed an increase in fluorescence intensity during aging, with protein largely concentrated in the cell bodies. From these studies we concluded that, compared to wild-type tau, both P301S and 3PO can accumulate significantly *in vivo* during aging, although the variants appear to be differentially distributed within the cells.

### P301S and 3PO tau form detergent insoluble aggregates in *C. elegans* neurons

**FIGURE 4:**
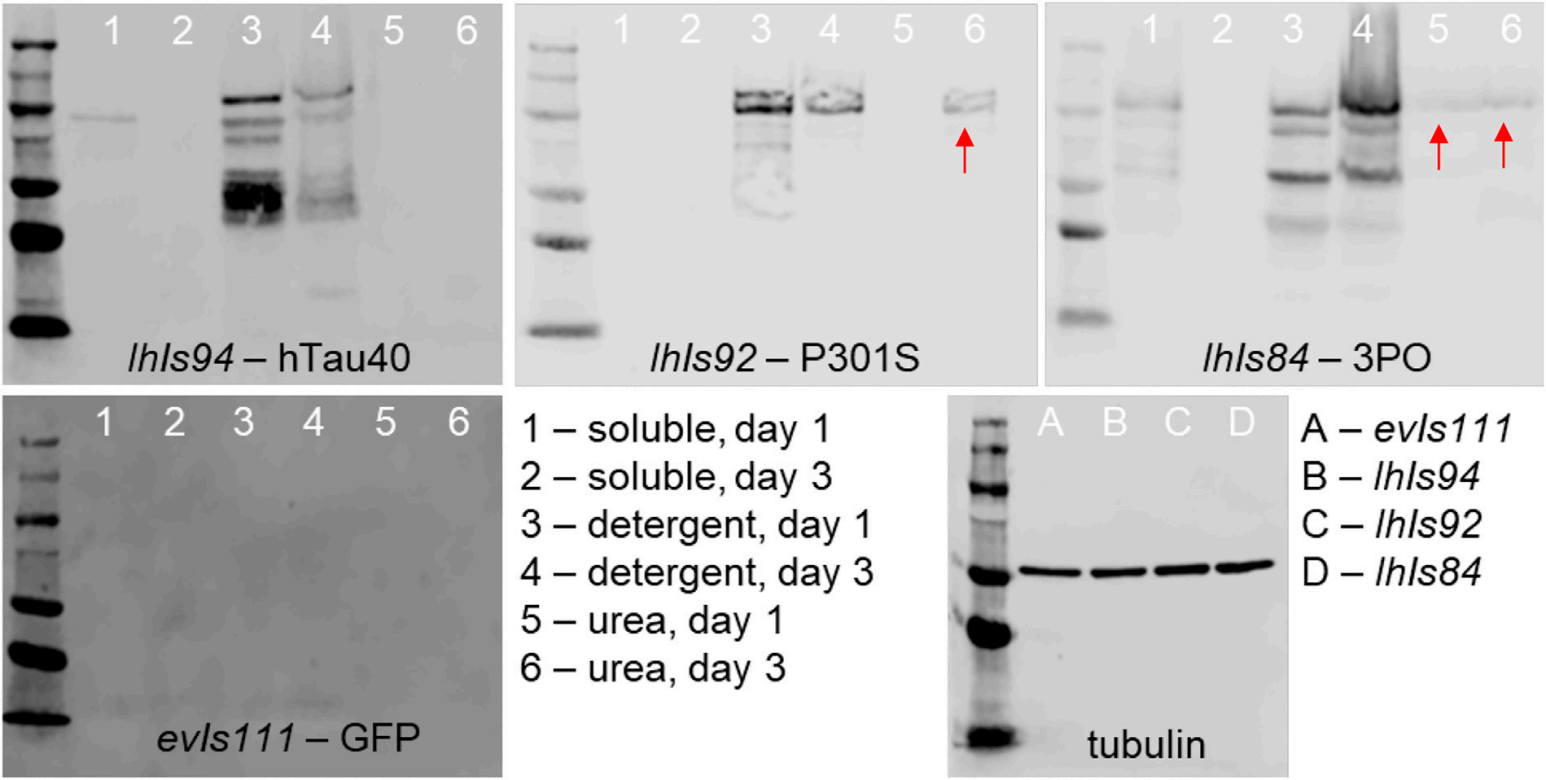
Serial biochemical extraction of aggregated tau. Protein was extracted from transgenic animals of the indicated genotype and day, then subjected to a series of extraction steps to collect the soluble (lanes 1 and 2), detergent soluble (lanes 3 and 4) and urea soluble (lanes 5 and 6) fractions. Fractions were separated by SDS-PAGE and detected using tau antibodies and chemiluminescence. The presence of tau in the urea fractions (red arrows) is indicative of aggregates that have formed. We found that tau is present in this fraction by day 1 of adulthood in 3PO expressing lines and by day 3 in P301S expressing lines. To the bottom right is a blot of extracts from day 1 animals of the indicated genotype of a control protein, tubulin, to demonstrate equal loading.

Previous work has shown that tau aggregates in *C. elegans* can be biochemically characterized via serial extraction (Kraemer et al., 2003). We isolated protein from our tau transgenic animals, or a non-tau control (*evIs111*), on days one and three of adulthood and used serial extraction (Materials and methods) to characterize the state of aggregation (Figure 4). Briefly, we separated proteins into the soluble (RAB buffer), detergent soluble (RIPA buffer) or detergent insoluble (Urea extracted) phases, and then performed SDS-PAGE and western blots.

We found that, as expected protein isolated from our non-tau animals had no immunoreactivity to tau antibodies on Western blot. The wild-type protein, hTau40, was present in either the soluble or detergent soluble phase, with no protein detectable in the Urea-extracted fraction. In contrast, the 3PO protein was detected in all three phases, as early as day one of adulthood. Interestingly, for the P301S line, we found that on day one protein was found only in the soluble and detergent soluble fractions. However, we detected P301S tau in the detergent-insoluble fraction from day three adults. Based on this pattern, we concluded that the P301S tau was forming detergent-insoluble aggregates in *C. elegans,* albeit more slowly than 3PO*.* We also were able to conclude that the 3PO protein was more aggressively aggregating, as would be expected from previous biochemical analyses (Iliev et al., 2006).

### Expression of aggregation-prone human tau reduces lifespan

To determine whether our tau expression lines were impacting the organisms we measured organismal lifespan. Several other tauopathy nematode models have shown that expression of tau can be detrimental using lifespan as a readout (Kraemer et al., 2003; Pir et al., 2016). This suggests that lifespan assays can provide information that helps us assess healthspan deterioration in the nematode.

**FIGURE 5:**
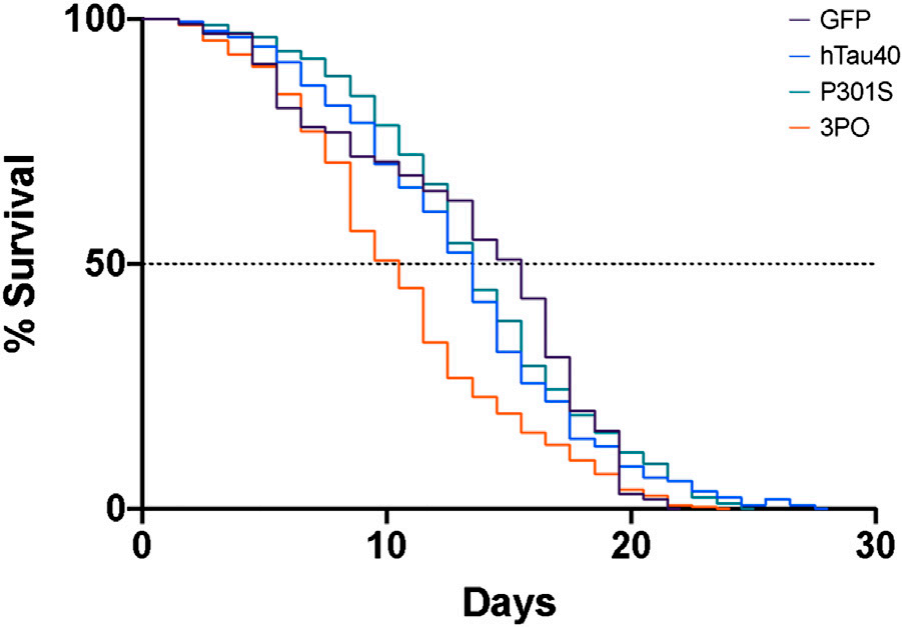
Expression of mutant tau in the nervous system induces premature lethality. A cohort of animals expressing GFP, hTau40GFP, P301SGFP or 3POGFP were counted daily for survival. We determined the day of 50% population survival (LD50). 3PO animals exhibit an LD50 of ∼8 days, P301S ∼11 days and WT tau of ∼12 days (3PO is significantly different from GFP, *p* < 0.001, log-Rank test). Each line represents 2–5 independent experiments.

We age-synchronized nematodes and plated a cohort of 50 (per replicate) L4 staged animals (day 0). We then proceeded to count the number of living animals every day (see materials and methods) until all had died (Figure 5). We found that 50% of hTau40 and P301S animals died by day 14, neither of which were significantly different from the no tau control. In contrast, 50% of the 3PO animals had died by day 10, demonstrating a reduced lifespan (Figure 5). We observed a sharp drop in the number of living animals around day seven, which correlated with the significant increase of 3POGFP levels on day five (Figure 3). Interestingly, the P301S, a disease-associated variant, did not appreciably cause a reduced lifespan, when compared to hTau40 or GFP alone, despite the increased accumulation observed. Together these argue that accumulation, by itself is not sufficient to impact lifespan, but that a pro-aggregation variant of tau can negatively affect organismal survival.

### Aggregation-prone tau induces changes in locomotion

**FIGURE 6:**
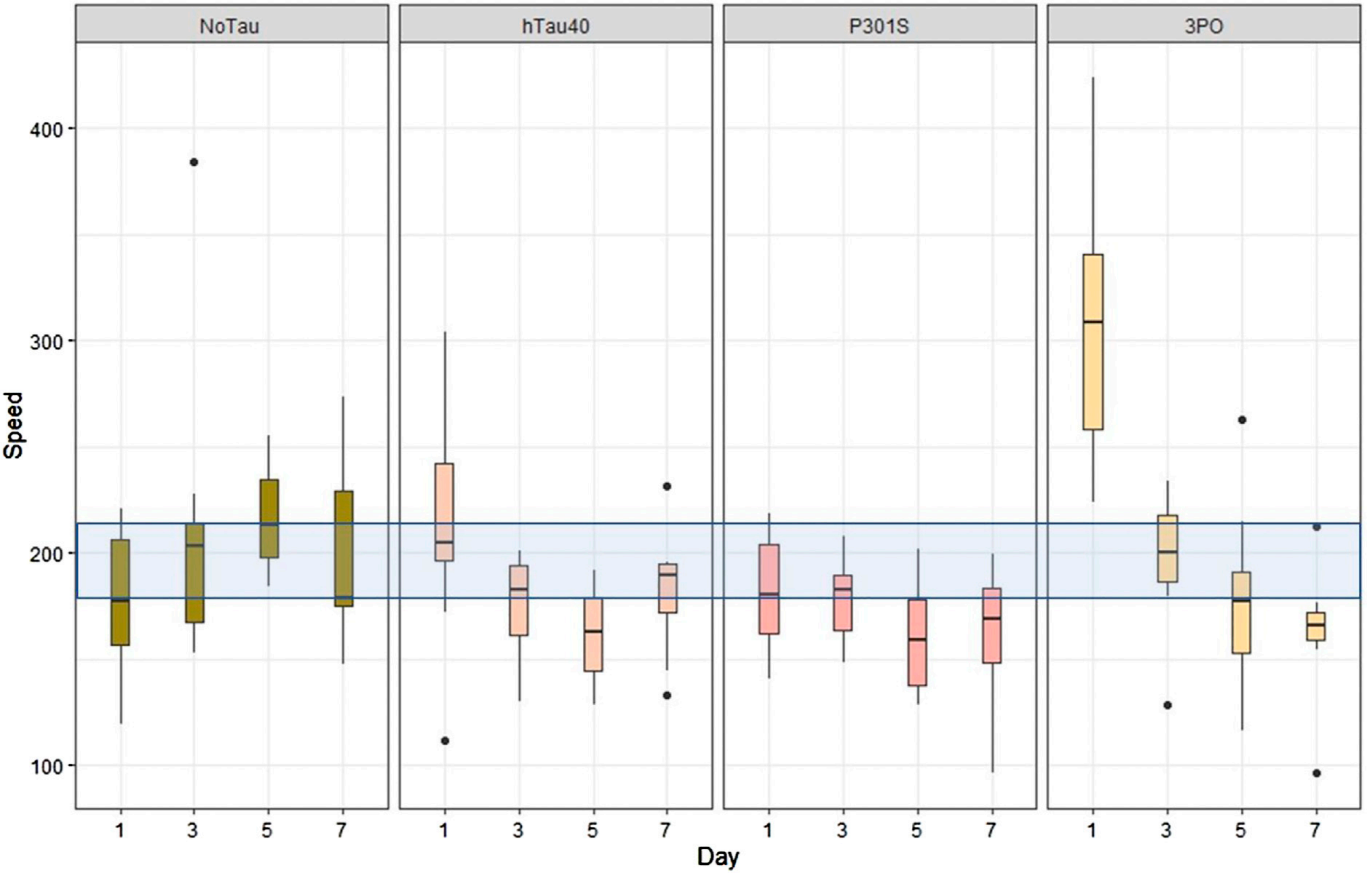
Expression of mutant tau affects locomotion. Animal motility was acquired by video microscopy and analyzed using tracking software (WormLab). When the speed of movement was quantified, the 3PO animals exhibited a hyperactive behavior on day one, but movement progressively slowed as the animals aged. Movement speed is plotted by day, with the blue transparent bar indicating the range of the mean speed of the control animals (*evIs111*) from all the days measured. The day one 3PO animals were significantly different from all the other lines on each day (*p* < 0.001). *N* = 10 for each transgene on each day.

The *C. elegans* locomotor system relies on both excitatory and inhibitory motorneurons to alternately induce muscle contraction or relaxation, resulting in sinusoidal crawling behaviour (Zhen and Samuel, 2015). Forward and backward locomotion are controlled by different classes of motorneurons, while turning behaviours can be initiated by sensory events. Thus, evaluating locomotion is a way to examine the ensemble function of the *C. elegans* nervous system. We acquired movies of transgenic animals crawling on NGM plates (see Materials and methods) and analysed these using tracker software (WormLab). The most obvious pattern to emerge was that the 3PO animals were hyperactive, compared to the no tau control, hTau40 or P301S animals, on day one of adulthood, but that their movement decreased progressively, on each day tested (Figure 6). From these analyses we concluded that the expression of the 3PO version of tau was impacting neuronal function in the early stages of adulthood.

### Human tau variants behave differently in the *C. elegans* ortholog of tau, ptl-1, mutant background

Tau is a microtubule-associated protein that binds tubulin and provides microtubule stability (Kadavath et al., 2015; Barbier et al., 2019). Thus, we asked whether there is an interaction between our expressed human tau and the *C. elegans* tau ortholog *ptl-1*. Previous reports suggest that human tau can rescue some, but not all, of the phenotypes observed in *ptl-1* mutants (Chew et al., 2013).

We crossed our tauGFP strains into the *ptl-1 (ok621)* background. The *ok621* mutation is a deletion that removes most of the *ptl-1* coding region, and the mutation has been reported to accelerate the onset of neuronal aging (Gordon et al., 2008; Chew et al., 2013).

**FIGURE 7:**
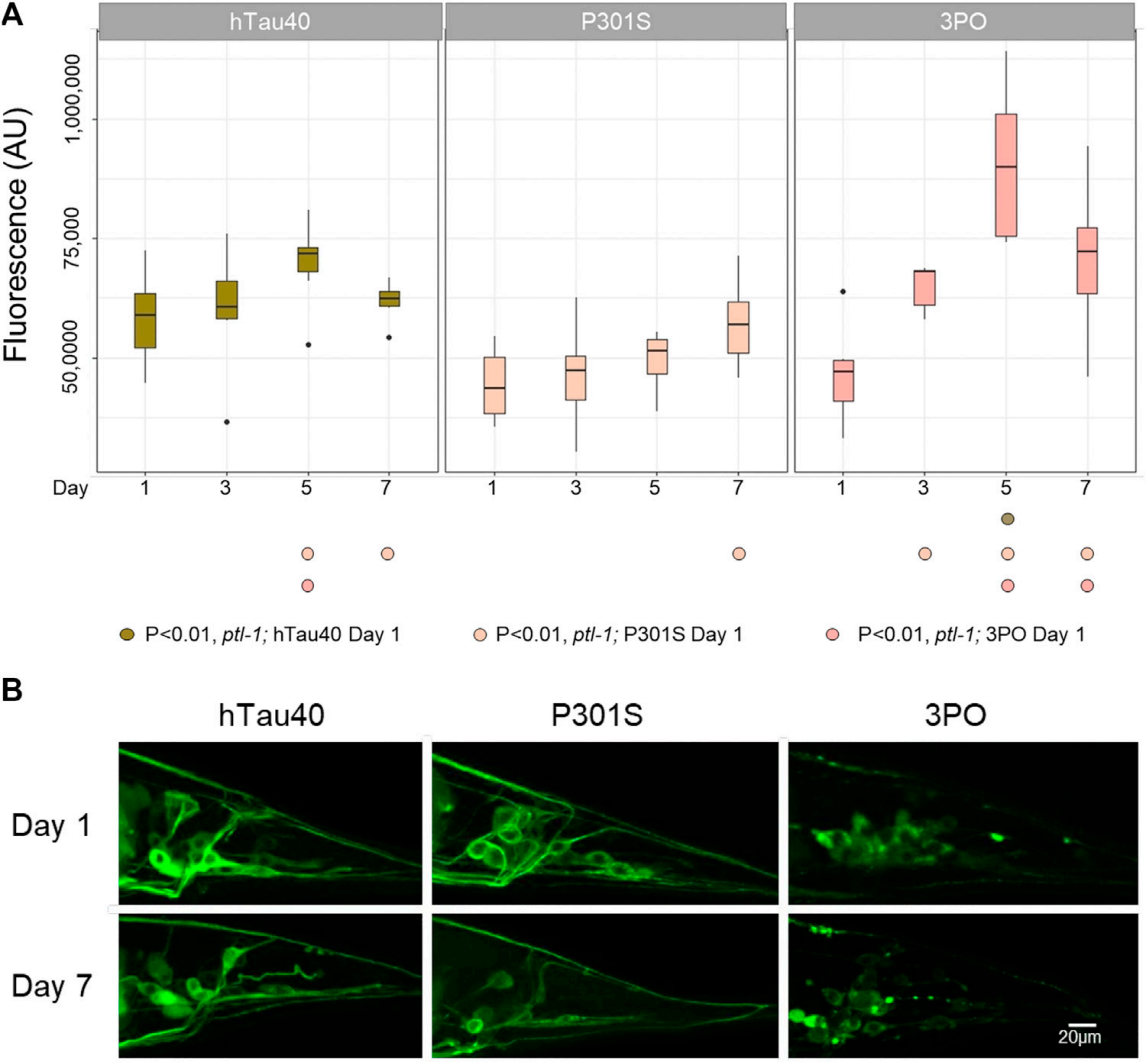
TauGFP variant accumulation in ptl-1 mutants. **(A)**. The *ok621* mutation reduced the accumulation of P301SGFP that occurred in wild-type background, but not the increased stability of 3POGFP. ANOVA (Day*Variant) with Tukey post-hoc comparison. *N* = 10 for each sample. **(B)**. Representative images of GFP accumulation in *ptl-1* mutants on days one and seven.

We again measured the total pixel intensity of tauGFP in the tail (Figure 7). Like the wild-type animals, the levels of hTau40 remained relatively consistent over the time observed. Interestingly, in animals lacking *ptl-1* the P301S variant did not display increased accumulation with aging. Rather, the levels stayed relatively constant over time. Qualitatively, the hTau40GFP and P301SGFP remained localized in neurons and axons in the *ptl-1* mutant animals. Thus, we hypothesize that the axonal accumulation of the P301S was dependent on the endogenous *ptl-1,* or that in the absence of *ptl-1* there are compensatory mechanisms that facilitate the clearance of the P301S protein. We continued to observe significant accumulation of 3POtau when *ptl-1* was absent, (Day 1 vs. Day 3 *p* < 0.0077, Day 1 vs. Day 5 *p* < 0.00001 and Day 1 vs. Day 7 *p* < 0.0004; Two-way ANOVA with Tukey’s correction). Overall, this provides further evidence for the differences in the accumulation of the protein variants, with the P301S affected by the loss of *ptl-1,* but the 3PO less so.

### *ptl-1* protects the animal from tau3PO effects

**FIGURE 8:**
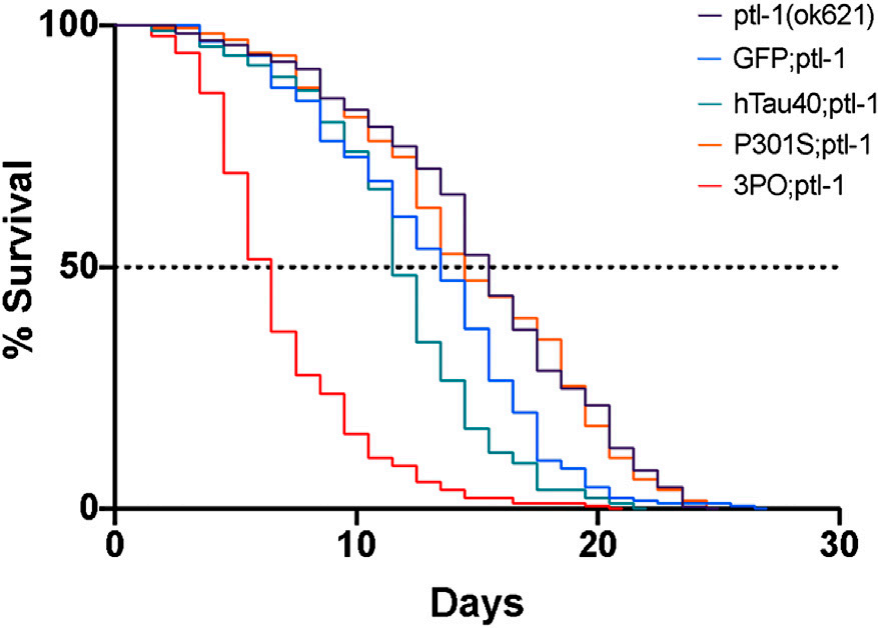
Lifespan of ptl-1 mutants expressing human tau. Animals were monitored daily for survival. All the lines expressing mutant tau died prematurely, compared to the *ptl-1(ok621)* single mutants (log-Rank test). We determined the LD50 as follows: 3POGFP; *ptl-1* ∼5 days, P301SGFP; *ptl-1* ∼15 days, hTau40GF; *ptl-1* ∼12 days, and *ptl-1(ok621)* ∼ 13 days. Curves represent 4 independently conducted experiments.

We repeated the lifespan assay in the *ptl-1* mutant background. There were no differences in the lifespans of wild-type or *ptl-1(ok621)* animals expressing GFP alone as a control (*p* = 0.18) (Figure 8). In the *ptl-1* background we observed a reduced lifespan due to hTau40GFP (*p* < 0.001) and 3POGFP animals (*p* < 0.001), while the P301SGFP lifespans were not different from *ptl-1* alone (Figure 8). It is not clear why the expression of wild-type human tau in the *ptl-1* mutant background was detrimental, while the P301S was not. However, the observation that the *ptl-1;*3POtau animals died more quickly than the expression of 3PO in the wild-type background suggested that the presence of the tau ortholog was beneficial to organismal survival.

### Tau adopts an aggregation-prone conformation detected by TNT1

**FIGURE 9:**
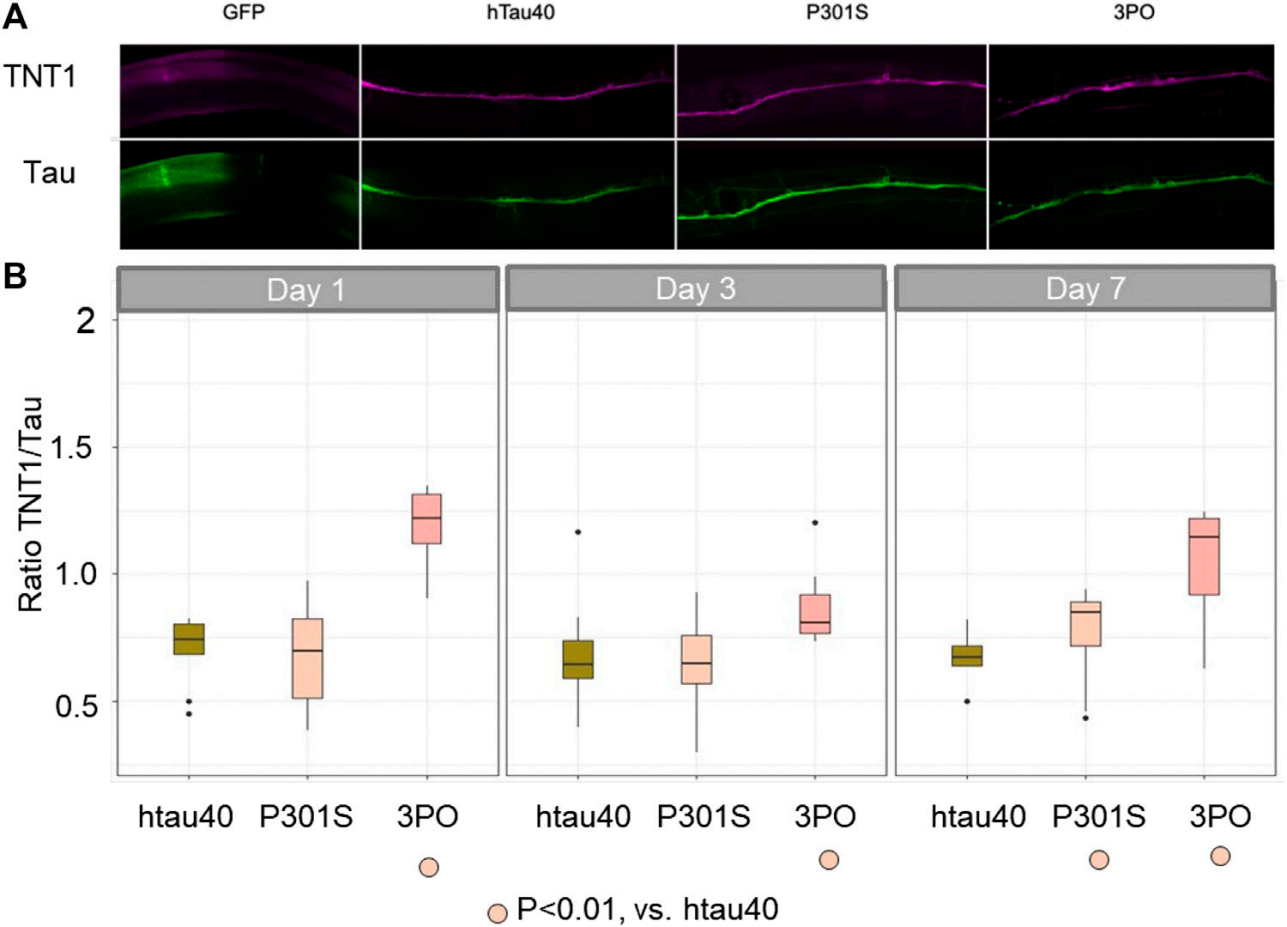
TNT1 staining indicates tau can adopt disease-relevant conformations in ***C. elegans*** We used the TNT1 antibody and A002401 antibody to stain animals for the early disease conformation aggregates compared to total tau. **(A)** Day one adult animals stained with TNT1 (magenta) and total Tau (green) antibodies. **(B)** We quantified the staining on days one, three and seven of adulthood (*N* = 10 per day per genotype), deriving the ratio of TNT1/Total tau. The 3PO lines had a higher ratio of pathogenic tau than hTau40 or P301S, which were not significantly different from each other on day one. By day seven the TNT1 immunoreactivity was significantly increased in the P301S animals compared to hTau40 (ANOVA, Tukey post-hoc comparison, *p* < 0.05).

Finally, to determine if the tau accumulation observed was relevant to the human disease we stained our transgenic lines using an antibody that preferentially binds to conformational changes in tau associated with tau aggregation (TNT1) (Combs et al., 2017) and an antibody that recognizes tau in general (A0021-A). We quantified the ratio of TNT1 intensity to total tau intensity in the ventral nerve cord of the animals during aging (Figure 9). We saw no staining in our negative controls (*evIs111,* GFP alone), indicating that the staining was specific to the tau-expression lines. We found that the ratio of TNT1/total tau was not significantly different between animals expressing the hTau40 or P301S on day 1, but there was a significantly higher ratio of TNT1 staining in the animals expressing the 3PO variant. As aging progressed the ratio of aggregated to total tau was stable in the hTau40 lines, but was consistently higher in the 3PO tau. By day seven we found a significant increase in the TNT1 immunoreactivity in P301S expressing animals. Thus, we concluded that the conformation of some of the tau species formed in the nematodes was like that formed in human disease early in the aging process and the ratio of aggregated tau increases in animals expressing 3PO or P301S.

## Discussion

### Expression of a gfp-tagged wild-type tau permits visualization of age-related changes

*In vivo* animal models are extremely valuable for understanding neurodegenerative conditions like Alzheimer’s disease and related disorders. While vertebrate models have significant utility in terms of their neuronal complexity and genetic relationship to humans, there are some drawbacks that can be better addressed using invertebrate models. For example, it is much easier to create new transgenic animals and, in general, phenotypes develop much more quickly. It is also simpler to cross these transgenes into animals with mutations in genes of interest that have been isolated in a common genetic background, thereby reducing potential confounds of genetic variation on the phenotypes observed.

To address some of the novel potential for invertebrate models we relied on the fact that *C. elegans* are optically transparent throughout the entire lifecycle, enabling us to visualize tau in living animals. Importantly, we observed that wild-type tau (hTau40), was well-tolerated by the animals at the DNA concentration we injected to create the transgenes. This contrasts with other *C. elegans* tauopathy models where over-expression of wild-type tau had a negative impact on survival (Kraemer et al., 2003). One difference could be the use of the 1N4R isoform in that study, while here we used the 2N4R isoform. However, the identification of expression levels in which the wild-type tau is non-pathological allowed us to observe the “normal” behaviour of tau inside an organismal nervous system during aging.

We did notice some differences between the accumulation in neurons of the ventral nerve cord and in the tail, where more of the tauGFP was observed in cell bodies. There are two potential explanations for this difference. First, the cells in the ventral nerve cord are primarily motor neurons, while the neurons observed in the tail are either sensory or interneurons, and thus, it is possible that tau accumulates differently in the various classes of neurons. Second, the morphology of the neurons is distinct, with the neurons in the tail having the cell body “between” the axonal and dendritic compartments, whereas the motor neurons have cell bodies that are somewhat lateral to the processes in the nerve cord (White et al., 1976, 1986). Thus, the observed differences may be more of a consequence of the morphology. Future experiments limiting tau to a subset of neurons should enable us to better distinguish which of these is more likely.

Interestingly, over the first 7 days of adulthood (approximately one-third to one-half of the animal’s lifespan) we did observe some differences in the intensity of GFP for wild-type tau across different cells. While this could simply be a product of differences in expression, it might also suggest that some neurons are more susceptible to tauGFP accumulation, as has been previously reported. In two different studies using mice, it appears that GABAergic neurons are more likely to accumulate neurofibrillary tangles (NFTs) and display deficits in activity (Andrews-Zwilling et al., 2010; Levenga et al., 2013; Najm et al., 2020).

Finally, it is important to note that we do observe immunoreactivity with an antibody that preferentially detects a disease-associated conformation of tau in post-mortem tissue from humans (TNT1). Thus, even in the short time frame of a few days, we find that in the *C. elegans* nervous system, tau adopts conformations similar to that of human disease. We followed this up by extracting tau from our transgenic animals using increasingly stringent buffers. We found evidence of detergent-insoluble tau only in 3PO and older P301S animals, but not hTau40. Thus, at least some of the TNT1 staining in these animals is indicative of a soluble form of tau. There is growing evidence in the field that larger aggregates may be protective and smaller oligomeric species more pathogenic, and thus it is interesting that we observe tau species detected by TNT1 that appear to be soluble.

### Tau variants can act differently from wild type

We evaluated two different tau variants, one, the P301S mutation, is a disease associated variant that has been linked to multiple tauopathies in humans, including frontotemporal dementia (FTD), corticobasal degeneration (CBD), and progressive supranuclear palsy (PSP) (Bugiani et al., 1999; Kametani et al., 2020). The second, 3PO, has been used both *in vitro* and *in vivo* to model a rapidly polymerizing version of tau that does not require an inducer molecule to initiate aggregation.

One of the results we observed was that, for the most part, the P301S variant behaved like wild-type tau in our models, with a few notable differences. Like wild-type tau, it was well-tolerated in its effects on lifespan and organismal motility.

Unlike the wild-type tau protein, P301SGFP appeared to be more stable, increasing in intensity over the time frame we observed, preferentially accumulating in nerve processes, and could aggregate into detergent-insoluble species by day three of adulthood. Some tau mutants, including the P301L variants can be resistant to cellular degradation, independent of their ability to aggregate (Caballero et al., 2018). Interestingly, the observed increased stability of P301S was dependent on the endogenous *C. elegans* MAP, PTL-1. Whether this indicates a physical interaction between the P301S and PTL-1, or that there are other compensatory changes in the *ptl-1* mutants that reduced P301S from accumulating needs to be further studied.

As would be expected, we observed significantly different consequences using the rapidly polymerizing variant. First, we confirmed biochemically that 3PO protein was forming aggregates, and that TNT1 immunoreactivity was increased in the 3PO-expressing animals. The 3POtau was more concentrated in cell bodies, and even in nerve processes appeared different from P301S or wildtype. These are consistent with previous work *in vitro* documenting the kinds of aggregates formed by 3PO compared to P301 mutants (Combs and Gamblin, 2012; Mutreja et al., 2019). Animals expressing 3POtau died sooner than expected, and the decline in organismal lifespan was around the time we begin to see an increase in overall tau protein levels, which is consistent with other nematode tauopathy models where increased expression of tau or tau variants was increasingly detrimental to the animals. We observed an interaction between 3PO and the loss of *ptl-1,* and others have previously reported an interaction between another rapidly polymerizing variant (ΔK280) and *ptl-1* (Ko et al., 2020).

Why was the P301S variant not particularly pathological in our model, given that other tau variants, including the oft-studied P301L have been shown to induce phenotypes? It is possible that the P301S takes longer to accumulate in a pathological form, and that we cannot observe that in a *C. elegans* lifetime (2–3 weeks). There may also be isoform differences in the aggregation properties (Mutreja et al., 2019). Thus, it will be interesting to study this in our system, asking whether 0N or 1N isoforms behave differently when the P301 residue is mutated.

**FIGURE 10:**
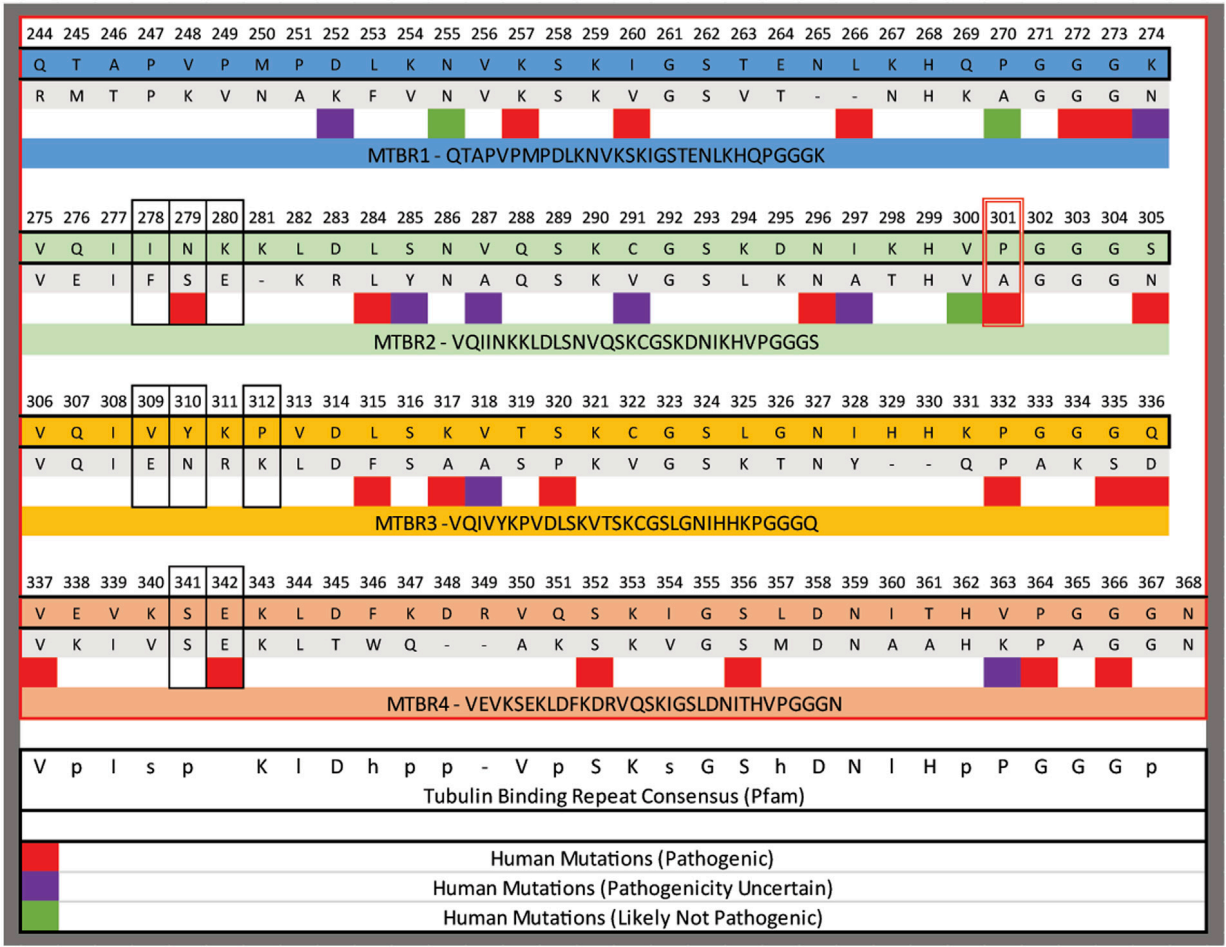
Alignment of the microtubule binding repeats (MTBR) from human tau and the C ***. elegan*** s PTL-1. The domains were aligned to each other using Clustal omega, numbering is from hTau40. The red box outlines the position of P301 mutated in the P301S lines and the black boxes outline residues modified in the 3PO variant. Below specific amino acids are regions that have been identified to in human populations, and the pathogenicity indicated by the colour code as indicated.

Another possibility is that the serine substitution is better tolerated by *C. elegans* neurons. The mutation is in the second microtubule binding repeat. In those repeats there is a highly conserved P-G-G-G-X motif that is predicted to enable folding of the MTBRs. And, in most mammalian tau proteins, the P is invariant. In contrast, when the MTBRs from the PTL-1 protein and human tau are aligned that proline residue is either an alanine or a proline (Figure 10). Thus, it is possible that that position has more flexibility inside the environment of *C. elegans* neurons. Finally, it is possible that the location within the neurons that we observe protein accumulation (cell bodies vs. processes) could be relevant.

There are differences in our results from previous work studying tau aggregation in *C. elegans.* Primarily, we do not observe any obvious cell death in the animals, at least in the time frame in which we focused. This contrasts with a recent study where the authors used a single-copy knock-in approach to study the effects of tau post-translational modification effects *in vivo* (Guha et al., 2020)*.* Other tauopathy models using over-expression of tau have noted cases of neuronal degeneration as well (Miyasaka et al., 2005; Kraemer and Schellenberg, 2007). There are differences in the isoforms being used in those studies, which motivates a more thorough investigation of isoform differences in tau-induced phenotypes.

### Amount of aggregated to non-aggregated tau impacts survival

Our quantification of the GFP fluorescence suggests that tau levels are being maintained. Since the activity of the *rgef-1* promoter is constant during the phase of adulthood we are analysing, the simplest interpretation for the stable levels of fluorescence is that tau is being produced and turned over at a constant rate. For the P301S and 3PO we observe an increase in fluorescence in an age-dependent manner, consistent with a reduced capacity to remove those proteins. However, only the 3POtau results in an impact on lifespan. There was significantly more TNT1 staining in animals expressing 3PO, suggesting more of the total protein was in a pathological conformation, consistent its generation as an aggressively pro-aggregating form of tau (Iliev et al., 2006). From this, we hypothesize that there is a threshold at which tau aggregates begin to impact organismal health. Together these results suggest that, like many other studies have suggested, tau-aggregates concentration must be maintained intracellularly to be toxic, and higher the concentration of tau deposition results in a worsening of the phenotype (Simón et al., 2012; Shigemoto et al., 2018). Whether that threshold is dependent just on the amount of aggregation, or the localization of the aggregation, or even other cellular factors remains to be determined.

### MAP proteins can protect against the effects of aggregated tau

PTL-1 is the *C. elegans* ortholog of human tau and other MAPs; it binds to tubulin and provides cytoskeleton support to the neurons and axons (Gordon et al., 2008; Chew et al., 2013). We found that a mutation ablating *ptl-1* function had multiple interactions with our tau expression lines. The most critical of these seemed to be that the loss of *ptl-1* resulted in a significant decrease in organismal lifespan in animals expressing 3POtau, which suggested that PTL-1 was protecting the animals from the consequences of the aggregated tau. Consistent with that effect, we found a reduction in the ability of the P301S variant to accumulate in axons when *ptl-1* was removed.

Overall, we have demonstrated that adding the GFP moiety to tau can help visualize tau in a living organism. Our current results suggest that aggregation, when confined to nerve processes may be better tolerated than when it is primarily in the somas. Using this system, we can begin to test genetic or environmental risk factors, trying to correlate the location and speed of aggregation to the changes in organismal lifespan. Ultimately, because of the simplicity of this system and rapidity with which phenotypes emerge, this model can be used to address many of the open questions in the field of tauopathies.

## Permission to reuse and copyright

Figures, tables, and images will be published under a Creative Commons CC-BY licence and permission must be obtained for use of copyrighted material from other sources (including re-published/adapted/modified/partial figures and images from the internet). It is the responsibility of the authors to acquire the licenses, to follow any citation instructions requested by third-party rights holders, and cover any supplementary charges.

## Data Availability

The raw data supporting the conclusions of this article will be made available by the authors, without undue reservation.
